# Transcriptional Regulation of Hepatic Autophagy by Nuclear Receptors

**DOI:** 10.3390/cells11040620

**Published:** 2022-02-10

**Authors:** Eun Young Kim, Jae Man Lee

**Affiliations:** 1 Department of Biochemistry and Cell Biology, Cell and Matrix Research Institute, School of Medicine, Kyungpook National University, Daegu 41944, Korea; key11@knu.ac.kr; 2 BK21 FOUR KNU Biomedical Convergence Program, Department of Biomedical Science, Kyungpook National University, Daegu 41944, Korea

**Keywords:** autophagy, macroautophagy, nuclear receptor, liver

## Abstract

Autophagy is an adaptive self-eating process involved in degradation of various cellular components such as carbohydrates, lipids, proteins, and organelles. Its activity plays an essential role in tissue homeostasis and systemic metabolism in response to diverse challenges, including nutrient depletion, pathogen invasion, and accumulations of toxic materials. Therefore, autophagy dysfunctions are intimately associated with many human diseases such as cancer, neurodegeneration, obesity, diabetes, infection, and aging. Although its acute post-translational regulation is well described, recent studies have also shown that autophagy can be controlled at the transcriptional and post-transcriptional levels. Nuclear receptors (NRs) are in general ligand-dependent transcription factors consisting of 48 members in humans. These receptors extensively control transcription of a variety of genes involved in development, metabolism, and inflammation. In this review, we discuss the roles and mechanisms of NRs in an aspect of transcriptional regulation of hepatic autophagy, and how the NR-driven autophagy pathway can be harnessed to treat various liver diseases.

## 1. Introduction

**Figure 1:**
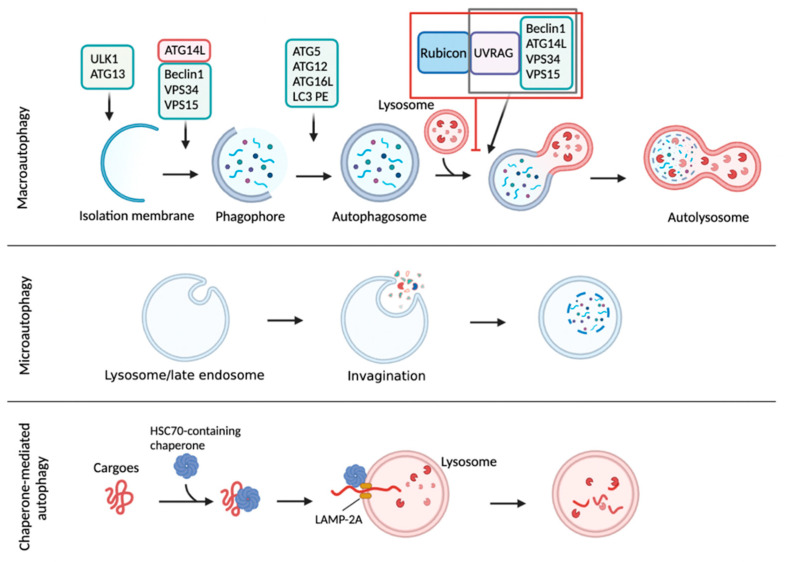
Autophagy is a catabolic process degrading cytoplasmic molecules, aggregated proteins, and infectious pathogens. There are three major types of autophagy: macroautophagy, microautophagy, and chaperone-mediated autophagy (CMA). Macroautophagy is initiated from an isolation membrane (also termed phagophore) to gather soluble materials and organelles for autophagosome formation. Autophagosomes fuses with lysosomes to form autolysosomes where cytoplasmic cargo molecules are finally degraded by lysosomal acidic hydrolases. Rubicon is a Beclin1-interacting protein involved in autophagy initiation and autophagosome maturation. Rubicon could directly bind to Class III PI3K-Beclin1-UVRAG complex for inhibition of autolysosome formation. In microautophagy, inward invagination of the lysosomal membrane or late endosome membrane engulfs small cytosolic components for their degradation. Lastly, CMA is mediated by a direct translocation of cargo proteins but not by the membrane reconstruction shown in macroautophagy or microautophagy. The cytosolic chaperone protein heat shock cognate 70 (Hsc70) and cochaperones recognize the specific pentameric peptide sequence (a KFERQ-like motif). The cytosolic proteins containing the KFERQ-like pentapeptide captured by Hsc70-cochaperone are translocated into the lysosome through a lysosomal associated membrane protein 2A (LAMP-2A) receptor on lysosomal membrane. This schematic diagram was created in BioRender.com (accessed on 20 January 2022).

Autophagy is a conserved intracellular degradation process delivering cytoplasmic materials to the lysosome. Unlike the ubiquitin-mediated proteasomal system (UPS) typically degrading short-lived proteins, autophagy involves a bulk process that degrades long-lived proteins, carbohydrates, lipids, and worn-out organelles, including ribosomes, peroxisomes, mitochondria, endoplasmic reticulum, and even nucleus [1,2,3,4,5]. In this way, autophagy is considered to promote nutrient recycling upon starvation and fulfill cellular energy demands [6]. There are three main types of autophagy: macroautophagy, microautophagy, and chaperone-mediated autophagy. Macroautophagy involves the formation of a double-membrane vesicle called an autophagosome (AP), which enables the engulfment of cytoplasmic materials and then fuses with lysosomes to generate autolysosomes (AL) where cargo molecules are degraded by lysosomal acidic hydrolases [1,2,3]. Microautophagy involves a direct engulfment of cytoplasmic cargo materials into lysosomes for their degradation [7,8,9]. In chaperone-mediated autophagy (CMA), cytosolic cargo proteins containing a specific targeting motif (KFERQ-like sequence) that is recognized by heat shock cognate 70 protein (HSC 70) are translocated into the lysosome through the lysosomal-associated transmembrane receptor 2A (LAMP-2A) for degradation [10,11] (Figure 1).

Nuclear receptor superfamily functions as transcriptional switches recognizing information of external changes and then delivering it to the genome, resulting in alterations of gene expression. They are either ligand-dependent or -independent transcription factors that play key roles in almost every mammalian physiology. Dysfunctions of nuclear receptor (NR) signaling pathways often become culprits that lead to many human diseases including liver diseases [12,13,14,15,16,17,18]. For a long time, autophagy regulation has been considered to exclusively occur in cytoplasm. This notion has been further supported by the findings that erythrocytes, enucleated cells, are still able to form autophagosomes [19,20]. Nevertheless, accumulated literature during the last two decades suggests that transcriptional and post-transcriptional events in the nucleus are also important for autophagy regulation. Since Christian de Duve initially described autophagy in rat hepatocytes upon the treatment of glucagon, a fasting-induced pancreatic hormone, the liver is the major organ where autophagy has been studied. Moreover, many nuclear receptors responding to presence or absence of nutrients play essential roles in the regulation of liver functions. In this review, we aim to summarize the transcriptional regulation of hepatic autophagy by NRs and to discuss how these regulations can contribute to the development of novel therapeutic strategies to treat liver diseases.

## 2. Overview of Autophagy

Autophagy is an evolutionarily conserved degradation process delivering intracellular cargo molecules to lysosomes. These cargos include glycogen, lipid droplets, aggregated proteins, membrane-enclosed organelles, and infected parasites [1,21,22]. Although most cells and tissues maintain a constant level of basal autophagy, numerous stimuli tremendously elevate autophagy activity. For example, autophagy can be markedly induced by physiological perturbations such as nutrient and growth factor deprivation, hypoxia, high temperature, high density conditions, and exercise. It is also activated by the endocrine hormones glucagon and FGF21, and diverse chemical reagents such as rapamycin, Torin, and AICAR. Many disease conditions, including cancer and muscle disease, are also known to have elevated levels of autophagy [23]. Although several types of autophagy have been reported based on different classifying criteria, macroautophagy is a primary form of autophagy, and it has been most intensively investigated so far compared with the two other forms of autophagy, called microautophagy and CMA (Figure 1). In most cells, there is a constitutive activity of macroautophagy, albeit at low levels, which contributes to constitutive turnover of cytosolic materials. This is considered as a basal autophagy activity. However, various cellular conditions, in particular nutrient deprivation, can markedly increase autophagy to stimulate the degradation of cytosolic substrates to generate energy and nutrient recycling. Two nutrient-sensitive kinases, mTORC1 and AMPK, rapidly respond to nutrient alterations and phosphorylate autophagy machinery proteins. In nutrient abundant status, mTORC1 phosphorylates two autophagy initiation proteins unc-51-like kinase 1 (ULK1) and ATG13, decreasing their autophagy initiation activity. In contrast, the absence of nutrients leads to inactivation of mTORC1 and subsequent activation of AMPK, which phosphorylates ULK1 and ATG13 on specific residues enhancing autophagy initiation. Next, in the nucleation step of autophagy, activated ULK1 complex increases the assembly of class III PI3K complex consisting of Beclin1, vacuolar protein sorting 15 (VPS15), VPS34, and ATG14L, to generate a local production of phosphatidylinositol 3-phosphate (PI3P) that is incorporated into the nucleated membrane of the autophagosome. In the maturation step, two ubiquitin conjugating systems are involved in autophagosome formation. The ATG12-ATG5-ATG16 complex is recruited to the autophagosome membrane and promotes a phosphatidylethanolamine (PE)-conjugation of microtubule-associated protein 1 light chain 3 (MAP1LC3, also known as LC3). This lipidated LC3 is required for the expansion of the autophagosome membrane. The resulting autophagosome then fuses with a lysosome, ultimately leading to the formation of an autolysosome where the autophagic cargos are degraded by lysosomal acidic hydrolases [24,25]. Rubicon could directly bind to Class III PI3K-Beclin1-UVRAG complex, resulting in the inhibition of autolysosome formation [26,27]. It is of interest to note that AL can also be made by an alternative pathway via a fusion of a lysosome with an amphisome, a vesicle created from a fusion between the AP and a late endosome (also known as multivesicular body) [28]. Degraded molecules within the AL are released into cytoplasm and recycled in the biosynthetic pathways to make new macromolecules or used for ATP production. In this way, autophagy is considered to link catabolism to anabolism [21,29,30] (Figure 1).

The degradative function of autophagy also plays a critical role in the intracellular quality control of many components by removing unfolded, misfolded, or aggregated proteins, or damaged organelles. Therefore, its cellular functions are very versatile, ranging from eliminating superfluous organelles to providing building blocks for lipid and protein synthesis, and energy supplementation to removing abnormal proteins for a quality control mechanism. Autophagy functions as a host defense mechanism by destroying invasive pathogens and subsequently presenting pathogen-derived antigens on the plasma membrane [1]. It has been suggested that excessive autophagy may trigger certain types of cell death including apoptosis and entosis [31,32]. Beyond its cellular functions, autophagy has a broad impact on mammalian pathophysiology including embryonic development, innate and adaptive immunity, neurodegenerative disease, cancer, heart disease and skeletal pathogenesis, ageing, and metabolic diseases [2,33,34,35,36]. On the contrary to a previous idea that autophagy may be a nonselective degradation process, numerous selective autophagies have been discovered depending on their cargo molecules and organelles. These include aggrephagy for aggregated proteins, glycophagy for glycogen, lipophagy for lipid droplets, ferritinophagy for iron-bearing ferritins, ribophagy for ribosome, pexophagy for peroxisome, reticulophagy for endoplasmic reticulum, mitophagy for mitochondria, xenophagy for virus and bacteria, nucleophagy for nucleus, and so forth [37,38].

Transcriptional regulation of autophagy was first observed in yeast where nitrogen starvation increased expression of autophagy gene *Apg8p*, the yeast homologue of mammalian LC3 [39]. During the last two decades, it has been demonstrated that many transcription factors, including NRs, induce the expression of autophagy genes, resulting in the enhancement of autophagy and degradation of unnecessary intracellular materials [40,41,42,43]. These findings paved the way for understanding autophagy regulation in the nucleus.

## 3. Overview of Nuclear Receptor Superfamily

As ligand-regulated transcription factors (TFs), NRs reside at the interface between environmental changes in cells and our genome, serving as an important linker between transcription and physiology. Thus, NRs play key roles in mammalian signaling because they can integrate diverse intra- and extracellular signals to initiate specific gene expressions for their relevant physiology. Owing to their unique characteristics in mammalian physiology, the activities of NRs are often affected by environmental stimuli that could produce ligands or sometimes modulate ligand production [14,44,45]. Moreover, NRs have a tremendous impact on most of mammalian physiology. Consequently, their dysfunctions are also associated with a broad range of human diseases, including metabolic diseases, cancer, immune disorders, cardiovascular diseases, and neurological diseases [45,46]. With a few exceptions, NRs usually consist of several domains: an N-terminal ligand-independent activation function 1 (AF1) motif, a highly conserved DNA-binding domain (DBD) with two zinc finger motifs, a flexible hinge domain, and a C-terminal less conserved ligand-binding domain (LBD) consisting of 12 α-helixes. Upon an agonist binding to the ligand-binding pocket of LBD, the helix 12 corresponding to ligand-dependent activation function 2 (AF2) motif undergoes a significant conformational change. This allows the exposure of a docking surface for coactivators that subsequently recruit more transcriptional machinery proteins to initiate the transcription of a given NR target gene [14].

**Figure 2:**
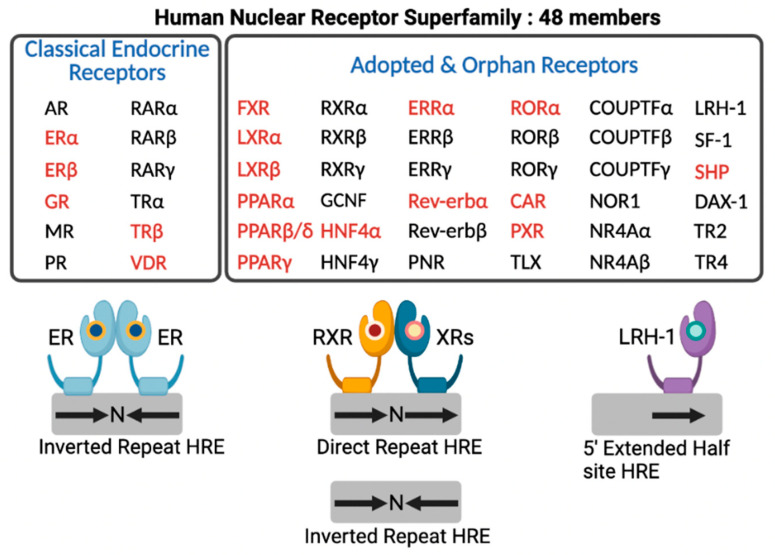
Human nuclear receptor superfamily. Human genome encodes 48 members of nuclear receptors (NRs). NRs are divided into two groups based on the source and type of their ligands. Classical endocrine receptors include steroid hormone receptors and RXR heterodimeric receptors. Adopted and orphan receptors include several receptors for dietary lipids, cholesterol derivatives, bile acids, phospholipids, and heme, and receptors with unknown ligands. The hormone response element (HRE) core sequence AGGTCA is represented by black arrows. N indicates any nucleotide between the half sites of HRE. Endocrine receptors usually bind as homodimers to palindromic DNA sequences (inverted repeats) separated by three nucleotides (IR3). Some NRs bind DNA as heterodimers with RXR to direct repeats separated by zero to six nucleotides (DR0-6) or to inverted repeats spaced by zero or one nucleotide (IR0-1). A few NRs interact with DNA as monomers to HRE containing a three-nucleotide 5’-extension. NRs shown in red colored letters are known to regulate hepatic autophagy, which is discussed in this review. This schematic diagram was created in BioRender.com (accessed on 20 January 2022).

In the human genome, there are 48 members of NRs that include classical endocrine receptors for steroid hormones, thyroid hormone, and fat soluble vitamins and their derivatives, adopted orphan receptors for fatty acids (FAs), phospholipids, cholesterol metabolites, bile acids (BAs), and heme, and orphan receptors whose ligands have not been discovered yet or may not exist at all [14] (Figure 2). Intensive research for nearly 40 years allows for enhancing our understanding of complex molecular mechanisms of an NR-driven transcriptional program. From these endless efforts, many NRs turn out to be valuable molecular targets for effective treatments in human diseases, which provide opportunities for developing better therapeutics with fewer side effects.

## 4. Classical Endocrine Receptors

### 4.1. Glucocorticoid Receptor (GR)

GR plays an important role in the regulation of genes involved in glucose homeostasis, inflammation, and stress response. GR activity can be potently modulated in response to glucocorticoids and synthetic ligands (e.g., dexamethasone, RU486, compound A, etc.), which are wildly used to treat inflammatory diseases. In addition to the roles of transactivation, GR has been shown to repress target gene expression via diverse mechanisms including transrepression and binding to non-canonical GR-binding site [47,48]. In particular, in the fasted liver, it has been shown that there are significant crosstalks between GR and other DNA-binding transcription factors and coactivators, including hepatocyte nuclear factor 4α (HNF4α), cAMP-responsive element-binding protein 1 (CREB), peroxisome proliferator-activated receptor α (PPARα), and peroxisome proliferator-activated receptor γ coactivator 1 α (PGC-1α) [49,50].

Regulation of hepatic autophagy by the modulation of GR functions has not been extensively studied in vivo so far. However, it has been reported that dexamethasone exposure to pregnant rats leads to inhibited proliferation and to dysplasia in offspring livers. Molecular mechanisms have been proposed that dexamethasone-activated GR increases FOXO1 mRNA and protein levels, which in turn induce hepatic autophagy in fetal livers [51]. In this context, the elevated autophagy activity by the GR-FOXO1 axis seems to be detrimental for normal development of fetal liver.

### 4.2. Estrogen Receptors (ERα & ERβ)

Estrogens play a pivotal role in the developmental processes of reproductive systems in females. Among two isoforms, ERα has been most studied in the liver. Estrogen binding enables dissociation of ERs from cytoplasmic heat shock protein 90 (HSP90), which is then translocated into the nucleus [52]. To control target gene expression, ERs bind to its response elements and then recruit coregulators. ERs have also been demonstrated to indirectly regulate target gene expression in a tether mechanism via other DNA-binding transcription factors such as members of the Forkhead box (FOX) family and activator protein 1 (AP-1) [53,54,55]. In the liver, the tethering mechanisms seem to be dominant due to the identification of AP-1 occupancies for ERα-binding sites [56]. ER activation with estradiol treatment has been reported to repress lipid biosynthesis and gluconeogenesis via the interaction with STAT3 [57,58].

Estrogen and ERs have been shown to have a strong gender bias in the autophagy regulation of Japanese medaka. Estrogen induces hepatic autophagy in female fish but suppresses it in male fish. This study further demonstrated that a specific ER isoform has a different mechanism for autophagy regulation. ERα induces hepatic autophagy via a hexokinase 2/AMPK/mTOR pathway, whereas ERβ2 increases autophagy in a Ca^2+^-dependent manner [59].

### 4.3. Thyroid Receptors (TRα & TRβ)

Thyroid hormone regulates cell growth, development, differentiation, and metabolism. T3, the major form of active thyroid hormone, can dynamically regulate transcription of target genes involved in lipid, glucose, and amino acid metabolism [60,61,62]. Among two isoforms, TRβ is predominantly expressed in the liver. TR forms a heterodimeric complex with RXR. Intriguingly, LXR and PPARα have been shown to compete with TR for binding to thyroid hormone response elements [63]. Without its agonist ligands, TR represses target gene expression by recruiting a corepressor complex containing nuclear receptor corepressor (NCoR) and histone deacetylase 3 (HDAC3) [64,65]. However, upon binding to thyroid hormones, conformational changes of TR lead to dissociation of corepressors and then recruit coactivators, resulting in increasing chromatin accessibility and target gene expression [66]. It has been suggested that TR activation controls lipid metabolism via diverse mechanisms, contributing to the improvement of non-alcoholic steatosis and inflammation in rodents [67,68].

Thyroid hormones are important for enhancing oxidative metabolism in the liver. The Yen laboratory has intensively studied to define roles of TR on hepatic autophagy. They have shown that T3 treatment drastically increases lipophagy, a selective autophagy for lipid droplets in hepatocyte- or hepatoma-driven cell lines and mouse liver. This allows released free fatty acids to be delivered to mitochondria for fatty acid oxidation (FAO) [69]. They have also reported that T3 treatment in human liver cells increases mRNA and protein levels of chromosome 19 open reading frame 80 (*C19orf80*) gene that promotes autophagy activity by facilitating a completion of autolysosome maturation [70]. Moreover, it has been demonstrated that T3 induces mitophagy, a selective autophagy for mitochondria through the oxidative phosphorylation (OXPHOS)-mediated reactive oxygen species (ROS) axis. ROS induction by T3 increases phosphorylation of 5’AMP-activated protein kinase (AMPK) via the activation of calcium/calmodulin-dependent protein kinase kinase 2, β (CAMKK2), which in turn leads to phosphorylation of ULK1, resulting in initiation of mitophagy. This study suggests that T3-mediated mitophagy is important not only for degrading damaged organelles but also for maintaining efficient OXPHOS [71]. An alternative pathway for TR-driven mitophagy induction has also been reported. There is a cooperative activity between TRβ and estrogen-related receptor α (ERRα) to regulate mitophagy. Many genes involved in mitochondrial metabolism are co-regulated by both NRs. It has been demonstrated that thyroid hormone-activated TRβ increases ERRα expression via the induction of *Pgc-1α* gene. ERRα now elevates ULK1 expression, which in turn leads to the activation of FUNDC1, a docking protein for LC3B-II. This complex pathway ultimately activates mitophagy in response to thyroid hormone treatment [72]. Finally, the Yen group has demonstrated that thyroid hormone levels significantly affect the development of hepatocellular carcinoma (HCC) through the modulation of aggrephagy, a selective autophagy for aggregated proteins. They have shown that thyroid hormone administration suppresses diethylnitrosamine (DEN)-treated HCC development in mice. Molecular mechanisms have been suggested that T3 increases death-associated protein kinase 2 (DAPK2) expression, which subsequently leads to phosphorylation of sequestosome 1 (p62/SQSTM1). This phospho-p62 promotes autophagic clearance of aggregated proteins, resulting in the alleviation of DEN-driven hepatocarcinogenesis [73]. Overall, thyroid hormone-activated TRβ has a profound impact on the induction of several types of hepatic autophagy including lipophagy, mitophagy, and aggrephagy. This may contribute to increased FAO and mitochondrial biogenesis, and decreased cancer promotion in the liver.

### 4.4. Vitamin D Receptor (VDR)

VDR is a key transcription factor for calcium homeostasis and skeletal health [74,75]. VDR is primarily activated by its endogenous ligands, an active form of vitamin D (1,25(OH)_2_D_3_) and bile acids lithocholic acid and its derivatives [76,77]. VDR forms a homodimer or heterodimer with RXR to control target gene expression along with coregulator complexes [78,79]. Defining a physiological role of vitamin D in the regulation of liver function has been limited due to the low expression levels of *Vdr* gene [80]. However, it has been elegantly demonstrated that there are robust *Vdr* expressions in other types of nonparenchymal cells, such as hepatic stellate cells (HSCs) and Kupffer cells, although hepatocytes express very low levels of *Vdr* [81,82]. In HSCs, VDR activation has been reported to potently repress TGFβ-induced profibrotic gene expression by antagonizing SMAD-dependent transcriptional programs, suggesting that targeting VDR with less calcemic ligands might be beneficial for the prevention and treatment of liver fibrosis [82].

It has been known that 1,25(OH)_2_D_3_ treatment significantly attenuates hepatic steatosis [83]. The Jiang laboratory has demonstrated that 1,25(OH)_2_D_3_ activates autophagy by inducing *Atg16l1* expression, which also has anti-inflammatory effects and improved lipid profiles [84]. Moreover, calcitriol administration was able to reduce ethanol-induced hepatotoxicity via the induction of AMPK/mTOR-mediated autophagy. VDR activation by calcitriol treatment increases the formation of APs and ALs, upregulates *Lc3b* and *Atg5*, and promotes degradation of p62, leading to mitophagy [85]. The studies of classical endocrine nuclear receptors for hepatic autophagy regulation are summarized in Table 1.

## 5. Adopted and Orphan Receptors

### 5.1. Farnesoid X Receptor (FXR)

FXR is a nuclear bile acid receptor and forms a heterodimeric complex with RXR, predominantly binding to inverted repeat 1 response elements of target genes. It plays a key role in bile acid homeostasis and its related lipid, glucose, and amino acid metabolism [86,87,88,89,90]. It has also been demonstrated that FXR is necessary for normal liver regeneration of wild-type mice, and for the beneficial effects of vertical sleeve gastrectomy on obese mice [91,92,93,94]. Several bile acids have been suggested as endogenous ligands for the regulation of FXR functions in enterohepatic tissues [95,96,97,98,99,100]. Natural antagonist and synthetic agonist ligands have also been reported and are currently intensively studied for clinical applications [101,102,103,104,105]. In these metabolic tissues, FXR has been suspected to be activated by returning bile acids in the process of enterohepatic circulation, indicating that FXR potently responds to a feeding status [88,106,107]. Therefore, FXR-mediated transcriptional programs have a significant impact on hepatic energy metabolism during postprandial periods [108,109].

The initial report that links FXR to autophagy regulation was that the treatment of GW4064, a synthetic FXR agonist, increases *Sqstm1* expression in mouse ileum, but not in mouse liver. The Guo laboratory has demonstrated that *p62* is a direct FXR target gene and suggested that this FXR-mediated p62 induction might provide a protective mechanism against tumorigenesis and inflammation [110]. The Ding laboratory has also shown that treatments of several bile acids induce accumulation of p62 proteins in mouse primary hepatocytes and livers, indicative of impaired autophagic flux. FXR activation in response to bile acids suppresses expression of the *Rab7* gene, whose protein is known to promote a fusion process of AP and lysosome to make AL [111]. Consecutively, two laboratories have simultaneously reported that hepatic FXR activation is sufficient for suppressing autophagy even in a fasted liver, and that FXR is required for autophagy suppression in a fed state of the liver. In these studies, FXR represses expression of many autophagy-related genes that can be upregulated by fasting-activated transcription factors such as PPARα and CREB [112,113,114]. Mechanistically, both FXR and PPARα can compete with each other to bind direct repeat 1 (DR1) response elements in the regulatory regions of autophagy-related genes. Moreover, FXR has also been shown to transrepress autophagy-related gene expression by disrupting a CREB-CRTC2 complex [112]. Consistent with these results, increased expressions of autophagy-related genes were observed in the liver of both *Fxr* and *Shp* double-knockout (*FS DKO*) as well as liver-specific *Fxr* and *Shp* double-knockout (*FS^LDKO^*) mice, suggesting that FXR acts as a negative transcription factor in this context [115]. In contrast to this, an acute ethanol treatment decreased expression of various autophagy-related genes and FoxO3 target genes in *Fxr* knockout (*Fxr^/−^*) mice. It has been suggested that increased AKT activity in the liver of *Fxr^−/−^* mice phosphorylates FoxO3, facilitating its cytoplasmic retention. These mechanisms seem to be associated with exacerbated hepatotoxicity and steatosis upon ethanol consumption [116]. Recently, a novel mechanism by which FXR suppresses autophagy in human cholestatic conditions has been reported. The Wagner laboratory has demonstrated that elevated bile acid levels in human cholestasis induce FXR-mediated autophagy impairments via the upregulation of *Rubicon*, which may inhibit a final fusion process between APs and lysosomes. Ursodeoxycholic acid (UDCA), a 7-OH epimer of CDCA, previously proposed as an FXR antagonist, improved human cholestasis by decreasing *Rubicon* expression, providing a novel therapeutic mechanism for cholestatic patients [117,118,119]. Overall, FXR activation potently inhibits hepatic autophagy by controlling expression of many autophagy-related genes, and autophagy induction by antagonizing FXR activity may be useful for treatment of certain liver diseases.

### 5.2. Peroxisome Proliferator-Activated Receptors (PPARα, PPARβ/δ, & PPARγ)

This subfamily contains three isoforms, and each member forms a heterodimer with RXR [120,121]. This receptor complex binds to peroxisome proliferation response elements (PPRE) of target genes [122,123]. Among PPREs, the most enriched response element is a direct repeat 1 (DR1) sequence [113]. PPARα was first identified due to its ability to induce peroxisome proliferation in response to Wy-14,643 [124]. Peroxisomes contribute to FAO, and their proliferation results in hepatomegaly and tumorigenesis in rodents, but not in humans [46,124]. Subsequently, two additional isoforms known as PPARγ and PPARβ/δ were discovered [125,126,127]. These NRs are activated by dietary fatty acids and their metabolic derivatives, and phospholipids, thereby serving as lipid sensors in our body. PPARα and PPARγ are mainly expressed in liver and adipose tissue, respectively, whereas PPARβ/δ is ubiquitously expressed throughout various tissues. Both PPARα and PPARβ/δ play an essential role in FAO and/or thermogenesis [124,125,128]. In contrast, PPARγ is famous for being a master regulator for adipogenesis and peripheral insulin sensitivity [127,129]. The importance of these receptors in pathophysiology is culminated by the fact that PPARα and PPARγ are molecular targets for lipid-lowering fibrate drugs and insulin-sensitizing thiazolidinedione (TZD), respectively [130,131,132]. Previous studies have also demonstrated that either overexpressing or targeting PPARβ/δ with a synthetic agonist ligand markedly improves exercise endurance, at least in rodents [133,134,135,136], indicating that harnessing this NR might be useful for the development of therapeutic strategies against metabolic diseases.

Transcription factor EB (TFEB) has been considered as a master regulator for lysosome biogenesis and autophagy and plays a key role in starvation-induced lipid metabolism such as FAO. In this context, PPARα is an important mediator of transcriptional outcomes governed by the TFEB-PGC-1α axis [137,138,139,140,141,142]. The compelling evidence that PPARα regulates autophagy has been reported by the Moore laboratory. They have shown that hepatic autophagy is coordinated by two nutrient-sensing NRs, a fasting-activated receptor PPARα and a nuclear bile acid receptor FXR [113,114]. PPARα is required for fasting-induced autophagy, and its pharmacological activation is sufficient for inducing autophagy, even in a fed state of mouse liver. PPARα activation increases hepatic autophagy via a direct induction of core autophagy-related genes. Moreover, it also leads to lipophagy [143,144,145]. A comprehensive cistromic analysis has revealed that a significant number of core autophagy-related genes are direct PPARα target genes in mouse liver. Overall, similar to TRβ, PPARα plays an important role not only in FAO and ketogenesis to provide ATPs and ketone bodies, but also in lipophagy to supply free fatty acids as substrates for FAO in a fasted liver [46,146,147]. The Kemper laboratory has also demonstrated that cAMP response element binding protein (CREB), a fasting-activated transcription factor, increases expressions of core-autophagy-related genes by binding to their promoter regions [112]. These results indicate that PPARα cooperates with CREB to control hepatic autophagy at the transcriptional levels. It would be very interesting to investigate the molecular mechanisms by which PPARα activation leads to lipophagy in liver.

PPARα activation with Wy-14,643 has been reported to suppress inflammation in mouse and human models of acute liver injury. This effect seems to be dependent on autophagy activity and *Atg7* gene. In particular, autophagy induction in macrophages in response to Wy-14,643 contributes to the protective effects on a lipopolysaccharide (LPS)-induced proinflammatory response [148]. Similarly, a mouse model of acute liver injury showed a reduced autophagy activity and downregulated *Pparα* gene expression. These effects can be reversed by inhibiting glycogen synthase kinase 3β (GSK3β) activity, suggesting that a GSK3β-PPARα axis in the liver is important not only for the induction of autophagy but also for subsequent hepatic protection in acute liver injury [149]. Consistently, PPARα activation reduces the anti-autophagic effects of miR-19a mimic and elevates LC3-II/LC3-I ratio and *Beclin-1* expression in models of acute liver injury [150]. Fibroblast growth factor 21 (*Fgf21*), a direct PPARα target gene in the liver, has pleotropic effects on diverse fasting-activated metabolic pathways including gluconeogenesis, FAO, and ketogenesis [151,152,153]. Fenofibrate, a hypolipidemic drug improves acetaminophen (APAP)-induced liver injury in wild-type mice but not *Fgf21* knockout mice, indicating that the beneficial effects of fenofibrate are at least in part dependent on *Fgf21*. In this context, fenofibrate increases LC3-II but decreases p62, resulting in the induction of hepatic autophagy. This mechanism seems to alleviate hepatotoxicity in APAP-treated wild-type mice [154]. Fasting-inducible FGF21 phosphorylates the Thr-1044 residue of the jumonji domain containing 3 (JMJD3/KDM6B) histone lysine demethylase via the activation of protein kinase A (PKA). Phosphorylated JMJD3 is then translocated into the nucleus where it interacts with PPARα, stimulating demethylation of the histone H3K27-me3. This event upregulates global autophagy-network genes including *Tfeb*, *Atg7*, *Atgl*, and *Fgf21*. This study has also suggested that upregulation of autophagy-related genes by PPARα depends on JMJD3 histone demethylase [155]. Zinc (Zn^2+^) has been known to stimulate hepatic lipid oxidation via upregulation of lipophagy. Zn^2+^ increases *Pparα* expression by promoting metal response element-binding transcription factor (MTF-1) to bind at *Pparα* promoter region. This in turn induces expression of key genes involved in autophagy and lipolysis [156]. Autophagy induction is important for the activation of hepatic c stellate cells (HSCs), which lead to liver fibrosis [157]. It has been shown that taurin supplementations reduces arsenic trioxide (As_2_O_3_)-induced HSCs activation via the inhibition of PPARα-mediated autophagy pathway [158]. Interestingly, autophagy also affects PPARα activity at the transcriptional levels. It has been reported that hepatic autophagy activity is essential for the functions of PPARα, particularly in a fasted liver. Liver-specific knockout mice for key autophagy genes *Atg5*, *Atg7*, or *Vps34* showed a compromised FAO and ketogenesis during a fasting period. These livers of knockout mice revealed marked accumulations of NCoR corepressor that inhibits PPARα-dependent transcription programs such as FAO and ketogenesis [113,159,160].

Cannabinoid-mediated antiproliferative effects in HCC cells have been found to be dependent on the induction of PPARγ. Knockdown experiments using siRNAs against PPARγ showed accumulated autophagy markers p62 and LC3B-II, indicating that PPARγ seems to be necessary for autophagy flux in cannabinoid-treated HCC cells [161]. Treatment of pioglitazone, a synthetic PPARγ agonist, ameliorated hepatic steatosis in high-fat diet (HFD) fed mice. In this experiment, it has been proposed that pioglitazone can increase hepatic autophagy along with increased cytosolic lipolysis and FAO probably by upregulating autophagy genes encoding ATG7, LC3, and LAL [162].

PPARγ has also been reported to alleviate arsenic As_2_O_3_-induced hepatotoxicity of rat offspring by suppressing ROS-mediated autophagy induction [163]. PPARβ/δ also seems to increase hepatic autophagy. PPARβ/δ activation reduced hepatic steatosis by the induction of autophagy-mediated FAO. Although detailed molecular mechanisms remain to be elucidated, synthetic agonists of PPARβ/δ can enhance AMPK activity that leads to inactivation of mTOR complex. This may trigger lipophagy, providing free fatty acids for mitochondria [164].

### 5.3. Liver X Receptor (LXRα & LXRβ)

LXRs play a pivotal role in cholesterol metabolism and inflammation. There are two isoforms, LXRα and β. LXRα is more abundant in liver and intestine but LXRβ is ubiquitously expressed. LXRs have oxidized cholesterol derivatives as physiological ligands [165,166]. Usually, LXR forms a heterodimer complex with RXR, and its more dynamic transactivation occurs upon the treatment of agonist ligands [63]. Global or hepatic knockout mice of LXRα fed with high cholesterol diets result in remarkable cholesterol accumulations in the liver [167,168]. Pharmacological LXR activation also markedly increases lipogenesis and fatty livers [169,170]. LXRα activation also influences mitochondrial functions by downregulating autophagy-related gene including *Atg4b* and *Rab8B*. It turns out that LXRα directly upregulates microRNAs let-7a and miR-34a that decrease stability of ATG4B and Rab8B mRNAs leading to autophagy inhibition in liver [171]. However, non-canonical activation of LXRβ in response to dendrogenin A (DDA) appears to have anticancer and chemopreventive effects on a cancer mouse model by inducing lethal autophagy. DDA-mediated LXRβ activation stimulates expression of autophagy genes such as *Lc3* and *Tfeb*, which are not observed by canonical LXR agonists. This may lead to new perspectives for cancer treatment [172].

### 5.4. Pregnane X Receptor (PXR) and Constitutive Androstane Receptor (CAR)

PXR is an essential NR for the defense mechanism against various foreign compounds called xenobiotics. PXR is activated by a variety of lipophilic xenobiotic molecules such as steroid derivatives, pesticides, herbs, prescribed drugs, endocrine disruptors, and other environmental toxic contaminants [173,174]. By sensing these xenobiotic chemicals and endobiotic molecules, PXR modulates expression of drug-metabolizing enzymes and transporters for detoxification in the liver and intestine. As with some of the other adopted orphan receptors, PXR also forms a heterodimer with RXR, which binds to PXR response elements (PXRE) such as a direct repeat 4 (DR4) or DR5 in the regulatory regions of target genes [175]. Mouse chromatin immunoprecipitation-sequencing (ChIP-seq) studies have revealed that PXR activation upregulates genes involved in cell proliferation and drug metabolism, but downregulates genes associated with amino acid and glucose metabolism [176].

PXR has also been shown to regulate hepatic autophagy. PXR positive cells showed upregulated p62/SQSTM1 but downregulated LC3-II, indicative of autophagy suppression. However, these were reversed in *Pxr* null cells. Pharmacological activation of PXR with rifampicin showed similar effects as shown in PXR positive cells. Mechanistically, p53-mediated induction of *Ampkβ1* gene can be inhibited by its physical interaction with PXR, thereby inhibiting ammonia-inducible hepatic autophagy [177]. Similarly, 18β-glycyrrhetinic acid (GA) as a promising hepatoprotective agent significantly decreased apoptosis and autophagic flux in the liver. GA has been shown to activate PXR, which suppresses an AP-lysosome fusion process and lysosomal stability [178]. Overall, PXR activation leads to autophagy suppression in the liver.

CAR is also intimately associated with drug metabolism and detoxification [179]. CAR mainly expressed in the liver plays an important role in energy metabolism [180,181]. CAR activation with a synthetic agonist TCPOBOP could form a heterodimer with RXR, and this complex typically binds to the phenobarbital-responsive enhancer module (PBREM) sequences for the upregulation of xenobiotic enzymes including cytochrome P450 CYP2Bs. Current studies regarding the role of CAR in autophagy have not been extensively explored. However, one study has shown that CAR activation by cadmium (Cd), a harmful heavy metal ion and a pollutant in rabbits, induces several *Cyp450* genes, resulting in ROS production. Increased ROS levels seem to activate excessive mitophagy and then cause liver damage [182]. Although little is known about CAR-mediated autophagy regulation, it should be an interesting research area to be explored in the future.

### 5.5. Hepatocyte Nuclear Factor 4 (HNF4α & HNF4γ)

HNF4α is known to be involved in hepatocyte differentiation and lipid metabolism [183,184]. Fatty acids and acyl-CoA thioesters have been proposed to act as endogenous ligands for HNF4α [185,186]. HNF4α activation regulates expression of a broad range of genes related to glucose, lipid, and inflammation via the formation of homodimer or heterodimer with an isoform HNF4γ. In addition, HNF4α can interact with other NRs to modulate hepatic glycolysis, lipid metabolism, and cholesterol homeostasis [187,188,189]. HNF4α has been shown to be upregulated in nonalcoholic fatty liver disease (NAFLD). Liver-specific *Hnf4α* knockout mice also developed fatty liver [190,191]. Recent studies have reported that autophagy activity is compromised in NAFLD owing to reduced ULK1 protein levels. In this context, miRNA Mir214-3p has been proposed to decrease the stability of *Ulk1* mRNA by a direct binding to its 3’UTR region. In contrast, HNF4α also directly binds to a specific regulatory region (−1643/−1534) of *Ulk1* gene and significantly upregulates its expression [192]. This study suggests that enhancement of *Ulk1* expression by either activating HNF4α or downregulating miRNA Mir214-3p probably alleviates fatty liver disease via the induction of autophagy activity.

### 5.6. REV-ERBα and REV-ERBβ

REV-ERBs play key roles in the negative feedback loop of the circadian transcriptional circuits in mammals. Hepatic REV-ERBs synchronize whole-body metabolism with food supplementations and environmental zeitgeber stimuli [193,194]. REV-ERBs have two isoforms REV-ERBα and REV-ERBβ expressed in various tissues including the brain and liver [180,195,196]. Heme has been proposed as an endogenous ligand of REV-ERBs and enhances their transrepression functions [197,198]. Unlike many other NRs, REV-ERBs act as a constitutive transrepressor of target gene expression by recruiting corepressors NCoR, SMRT, and HDCA3 [193,194]. Previous studies have also revealed that there is a direct competition between REV-ERBs and RORs to bind the same response elements of circadian genes [199,200].

It seems likely that there is a crosstalk between circadian rhythm and autophagy activity. Circadian rhythm has been shown to regulate autophagy via REV-ERBα. The number of APs and ALs are daily rhythms in zebrafish liver. In accordance with this, autophagy-related genes are significantly upregulated in the *Rev-erbα* mutant fish. Among them, *Ulk1* turns out to be a direct REV-ERBα target gene. Fasting also alters the expression of clock genes and autophagy-related genes in other peripheral organs [201]. Intriguingly, autophagy also affects circadian rhythms in the liver. Circadian proteins BMAL1, CLOCK, REV-ERBα, and CRY1 are targeted to lysosomes for their degradation. In particular, CRY1 proteins contain two LC3-inteacting region motifs (LIR) that facilitate their macroautophagy-mediated degradation. Since CRY1 acts as a negative transcription factor of gluconeogenesis, autophagy-mediated CRY1 degradation may contribute to the maintenance of blood glucose levels during fasting [202].

### 5.7. Retinoic Acid Receptor-Related Orphan Receptors (RORα, RORβ, & RORγ)

RORs have three major isoforms, RORα, RORβ, and RORγ. Among them, RORγ and RORγ are particularly important for coordinating the circadian rhythm, which affects lipid metabolism and inflammation in the liver [203]. Unlike REV-ERBs, RORs in general function as transcriptional activators. As mentioned above, RORs share DNA binding motifs of circadian clock genes, including *Bmal1*, with REV-ERBs. Because of this, RORα displays opposite circadian expression patterns of target genes compared with those of REV-ERBs [204]. Although many studies have been done to understand roles of RORs in circadian rhythm and metabolism, little is known that RORs regulate hepatic autophagy. A recent study has reported that RORα might be involved in autophagy regulation by controlling the acidity of lysosomes. RORα-deficient mice showed defects of autophagy flux in the liver. These defects in RORα deficient livers seem to be due to low expression levels of *Atp6v1g1* gene encoding a component of the peripheral stalk of v-ATPase [205]. Because v-ATPase controls lysosomal pH, downregulated *Atp6v1g1* expression by hepatic RORα deletion results in autophagy impairments. This suggests that RORα might oversee lysosomal acidification and an autophagy flux at the transcription level.

### 5.8. Estrogen-Related Receptor (ERRα, ERRβ, & ERRγ)

ERRs are orphan NRs that contribute to a variety of cellular metabolism to maintain energy homeostasis. There are three isoforms, ERRα, ERRβ, and ERRγ. Their hepatic functions have been studied in various conditions such as alcohol and lipid metabolism, bile acid synthesis, gluconeogenesis, and iron metabolism [206,207,208]. Until now, its endogenous ligand has not been identified. Among three ERR isoforms, ERRα is ubiquitously expressed and is required for both mitochondrial biogenesis and FAO [209,210]. ERRs are able to bind to ERR response element (ERRE) as a monomer or dimer of two ERRs [210]. ERRα enhances its transactivation activity by recruiting potent coactivators such as PGC-1α/β [80]. In terms of autophagy regulation, ERRα has been shown to be induced by AMPK or SIRT1 activation, which facilitates autophagosome formation. ERRα also increases expression of *Atg* genes containing ERREs in macrophages [211]. As described earlier, ERRα cooperates with TRβ1 to regulate many genes involved in mitochondrial metabolic pathway. ERRα seems to be required for T3-mediated mitochondrial biogenesis, fission, and mitophagy. For example, increased *Ulk1* expression by T3 is mediated by ERRα and activated ULK1 in turn promotes interactions between a docking receptor FUNDC1 and LC3B-II to induce mitophagy. Taken together, the TR-ERRα axis leads to a mitochondrial clearance via a ULK1-FUNDC1 pathway [72].

### 5.9. Small Heterodimer Partner (SHP)

SHP is an atypical orphan NR due to its absence of DNA-binding domain and is predominantly expressed in the liver and intestine, where it plays an essential role in bile acid biosynthesis [212,213,214]. Although synthetic chemical ligands have been developed, any endogenous ligand has not been reported yet [215]. Because of its unique structure, SHP cannot bind to DNA directly. Therefore, SHP functions as a corepressor and interacts with other NRs to control target gene expression. Three modes of action mechanisms have been proposed. First, SHP inhibits coactivator recruitment to DNA-binding transcription factors via a direct interaction. Second, SHP recruits more corepressor proteins. Lastly, SHP inhibits DNA binding of other transcription factors [215]. Recent studies have found that SHP and FXR cooperates to regulate the expression of autophagy-related genes such as *Atg7* and *Atg12* to restore autophagic flux in the liver [115]. SHP-mediated epigenetic regulation of hepatic autophagy has also been reported [216]. Hepatic autophagy is maintained by two-layered transcriptional programs via a sequential action of two NRs FXR and SHP. FXR first suppresses hepatic autophagy, which is then sustained by SHP in a fed state. To do this, SHP represses the expression of autophagy-related genes by recruiting histone demethylase LSD1, a repressive histone modifying enzyme in response to FGF19 [216]. In a fed or FGF19 treated condition, hepatic SHP recruits LSD1 and then disrupts CREB-CRTC2 complexes, resulting in reduced expression of CREB-target autophagy related genes such as *Tfeb*, *Atg3*, *Atg7*, and *Atg10*. This downregulation of autophagy genes seems to suppress hepatic autophagy. In HSCs, autophagy activation is necessary for the induction of profibrogenic gene expression. Consistent with its role in hepatocytes, SHP also inhibits autophagy in HSCs, which may be beneficial for the prevention of liver fibrosis. *Shp* knockdown experiments showed increased expression of fibrotic-related genes such as α-*SMA*, *collagen I*, and *TIMP1* [217]. Because of autophagy suppression by SHP in HSCs, further investigation should be helpful for understanding the role of SHP in liver fibrosis. The studies of adopted and orphan nuclear receptors for hepatic autophagy regulation are summarized in Table 2.

## 6. Conclusions

**Figure 3:**
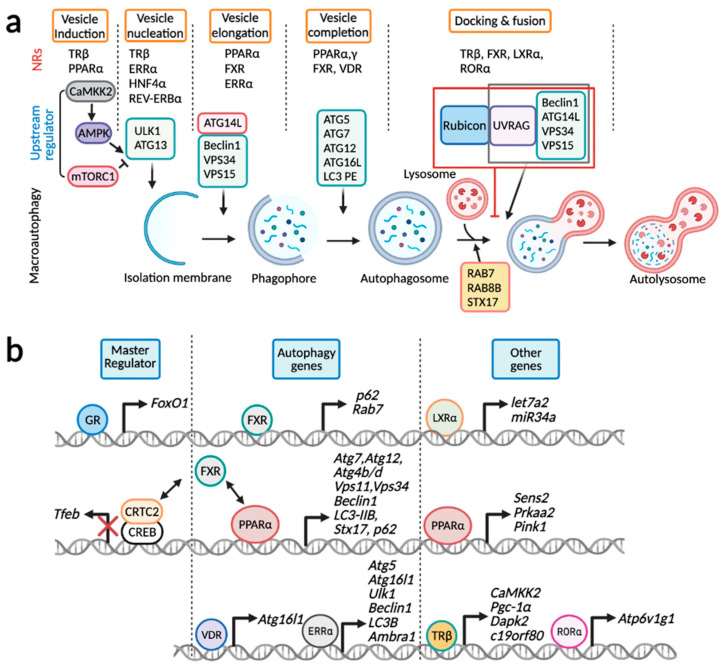
Transcriptional control of hepatic autophagy by nuclear receptors. (**a**) Each step of macroautophagy is typically controlled by multiple NRs: vesicle induction (TRβ and PPARα), vesicle nucleation (TRβ, ERRα, HNF4α, and REV-ERBα), vesicle elongation (PPARα, FXR, and ERRα), vesicle completion (PPARα, PPARγ, FXR, and VDR), and docking and fusion (TRβ, FXR, LXRα and RORα). (**b**) GR and FXR regulate expression of genes encoding master transcription factors FoxO1 and TFEB. PPARα, ERRα, VDR, and FXR control genes involved in core autophagy-related genes. FXR typically downregulates these core autophagy genes via the disruption of CREB-CRTC2 complex or competition with PPARα. Lastly, LXRα increases expressions of miRNAs let7a2 and miR34a that in in turn downregulate transcripts of *Atg4b* and *Rab8b* genes. PPARα and TRβ increases expressions of genes associated with either upstream regulators (Sestrin2, AMPK, CaMKK2, DAPK2) or PINK1, a critical kinase for mitophagy. RORα induces *Atp6v1g1* gene encoding a subunit of v-ATPase, which is critical for lysosomal acidification. This schematic diagram was created in BioRender.com (accessed on 5 February 2022).

In the last two decades, mounting evidence strongly suggests that hepatic autophagy can be controlled in the nucleus. Although many transcription factors, including NRs, have been identified for autophagy regulation, we still do not fully understand its physiological and pathological significance. Nevertheless, it is very likely that transcriptional control of a significant number of autophagy genes acts in concert with their post-translational regulation to advance the exquisite coordination of autophagic flux, in particular during long-term starvation or chronic stresses. Certainly, major proteins involved in autophagy machinery and cargo receptors themselves undergo autophagy-mediated degradation. In addition to this, lysosomes are also consumed in the process of autolysosome formation. Therefore, the transcriptional upregulation of lysosomal and autophagy-related genes could be a robust compensatory response against depletion of corresponding proteins during autophagy. Intriguingly, nuclear events of autophagy regulation are not limited to the transcriptional levels, but also involve post-transcriptional levels, highlighting complex layers of autophagy regulation (Figure 3).

Each step of macroautophagy is regulated by multiple NRs: TRβ and PPARα for vesicle induction, TRβ, ERRα, HNF4α, and REV-ERBα for vesicle nucleation, PPARα, FXR, and ERRα for vesicle elongation, PPARα, PPARγ, FXR, and VDR for vesicle completion, and TRβ, FXR, LXRα, and RORα for docking and fusion (Figure 3a). It is of interest to note that PPARα, FXR, ERRα, and TRβ engage in multiple steps of macroautophagy by regulating expression of key genes encoding upstream regulators, and autophagy machinery proteins for vesicle nucleation, elongation, completion, and docking and fusion. By contrast, it seems likely that a certain NR regulates specific sets of genes involved in the distinct step of macroautophagy. GR and FXR regulate expression of genes encoding FoxO1 and TFEB, master transcription factors for autophagy gene regulation (Figure 3b). Intriguingly, in addition to the inductions of genes *p62* and *Rab7*, FXR dynamically suppresses a variety of autophagy-related genes via either the disruption of CREB-CRTC2 complex or a direct genomic competition with PPARα for binding to DR1 sites. Expressions of core autophagy-related genes are primarily increased by PPARα, ERRα, and VDR. Genes encoding upstream regulators were also controlled by PPARα and TRβ. LXRα indirectly downregulates *Atg4b* and *Rab8b* transcripts by the inductions of miRNAs let7a2 and miR34a. Lastly, RORα also indirectly promotes macroautophagy by increasing lysosomal acidification through the upregulation of *Atp6v1g1* gene encoding a subunit of v-ATPase. Overall, NRs directly and indirectly control transcription or transcripts of autophagy-related genes via various mechanisms including transactivation, transrepression, recruitments of chromatin remodeling proteins, and miRNAs.

NRs have been attractive molecular targets to treat liver diseases because of their tight connections to the regulation of various metabolic pathways. Thus, in addition to affecting diverse aspects of liver biology, targeting hepatic NRs has been shown to actively control autophagy in various cell types such as hepatocytes, HSCs, Kupffer cells, and hepatoma cell lines. These modulations of autophagy activity in a cell-type specific manner can be very useful for developing novel therapeutics with less side effects. Moreover, it would be of importance to discover novel NR target genes encoding key proteins, miRNAs, and long non-coding RNAs (lncRNAs), which can affect post-translational modifications of core autophagy machinery proteins or become cargo receptors themselves to modulate selective autophagy. For the next decade, it will be a very exciting period to understand whether diverse NRs also regulate a specific type of selective autophagy in a cell type-specific manner in the liver, and if these regulatory mechanisms can be harnessed to fight metabolic liver diseases.

## Figures and Tables

**Table 1:** Classical endocrine nuclear receptors coordinate hepatic autophagy.

NRs	Ligand/ Activator	Study Model	Autophagy	Mechanism of Action	Refs.
GR	Dexamethasone	Rat fetal liver	↑	GR activation ↑ → *FoxO1* gene ↑ → autophagy- related genes ↑ → proliferation of fetal liver ↓	[51]
ERα	E2	Japanese Medaka fish	↑ (Female) ↓ (Male)		[59]
	−		↑	HK2 ↓ → AMPK ↑ → mTOR ↓ → ULK1 ↑	
ERβ2	E2		↑	Ca^2+^ dependent manner	
TRβ	T3	HepG2, AML12, Hep3B, Hur7, mouse liver, human liver cells	↑	*C19orf80* gene ↑ → AL ↑ → lipophagy↑ → FFA ↑ → FAO ↑	[69,70]
		HepG2, mouse liver	↑	OXPHOS ↑ → *Camkk2* gene ↑ → ROS ↑ → AMPK phosphorylation ↑ → ULK1 phosphorylation ↑ → mitophagy ↑	[71]
		HepG2, mouse liver	↑	TRβ → *Pgc-1α* gene ↑ → *Errα* gene ↑ → *Ulk1* gene ↑ → FUNDC1-LC3B-II → mitophagy ↑	[72]
		DEN-treated HCC in mice	↑	*Dapk2* gene ↑ → p62 phosphorylation ↑ → aggrephagy ↑	[73]
VDR	1,25(OH)_2_D_3_	HepG2, HFD-fed mice livers	↑	*Atg16l1* gene ↑	[84]
	Calcitriol	LO2, HepG2, *Vdr^−/−^* mice, mouse hepatocytes	↑	AMPK↑ → mTOR ↓ → LC3B-II & ATG5 ↑ AP & AL ↑ → p62 ↓ → mitophagy ↑	[85]

E2, Estrogen; AMPK, AMP-activated protein kinase; mTOR, mammalian target of rapamycin; HK2, hexokinase 2; ULK1, unc-51-like kinase 1; FFA, free fatty acid; FAO, fatty acid oxidation; ROS, reactive oxygen species; CaMKK2, calcium/calmodulin dependent protein kinase kinase 2; FUNDC1, Fun14 domain containing 1; DAPK2, death associated protein kinase 2; HCC, hepatocellular carcinoma; HFD, high-fat diet; ↑, increase; →, promote; ↓, decrease.

**Table 2:** Adopted & orphan nuclear receptors coordinate hepatic autophagy.

NRs	Ligand/ Activator	Study Model	Autophagy	Mechanism of Action	Refs.
FXR	GW4064	Mouse liver/ileum	↓	*p62* gene ↑ → tumorigenesis ↓ & inflammation ↓	[110]
		Mouse liver	↓	Autophagy flux ↓ Autophagy related genes ↓ Competition with PPARα for DR1 binding Disruption of a CREB-CRTC2 complex	[112,113]
	Bile acids	*Fxr^−/−^* liver, primary mouse hepatocytes	↓	*Rab7* gene ↓ → AP-lysosome fusion ↓ → p62 ↑ → autophagy flux ↓	[111]
	−	*FS DKO* mice *FS LDKO* mice	↑	Autophagy-related genes ↑	[115]
	OCA	Human cholestatic liver	↓	*Rubicon* gene ↑ → AP & lysosome fusion ↓ → AL ↓	[118]
	UDCA		↑	*Rubicon* gene ↓ → AP & lysosome fusion ↑ → AL ↑	
	EtOH	*Fxr^−/−^* liver	↓	FoxO3-mediated autophagy-related genes ↓ → hepatotoxicity ↑ → steatosis ↑	[116]
PPARα	Wy-14,643 GW7647	Mouse liver, AML12	↑	PPARα competes with FXR to bind to DR1 biding site of autophagy-related genes	[113]
	Wy-14643	Macrophage, Acute liver injury (LPS)	↑	PPARα-induced autophagy ↑ → *Beclin1* gene ↑ → LC3-II/I ratio ↑ → *miR-19a* gene ↓ → inflammation ↓ → acute liver injury ↓	[150]
	Fenofibrate	APAP-liver injury	↑	*Fgf21* gene ↑ → LC3-II → p62 ↓ → APAP-liver injury ↓	[154]
	Fasting	Mouse liver	↑	Fasting → *Fgf21* gene ↑ → PKA activation ↑ → JMJD3 phosphorylation ↑ → PPARα-mediated autophagy-related genes ↑	[155]
	Zn^2+^	Yellow catfish liver	↑	Zn^2+^ → MTF-1-mediated *Pparα* gene ↑ → autophagy- related genes ↑ → lipophagy ↑	[156]
	Taurin	HSC in mouse liver	↑	PPARα-mediated autophagy-related genes ↓ → arsenic trioxide-induced HSC activation ↓	[158]
PPARβ/δ	GW501516	Obese mouse liver Aged mouse liver HepG2, primary mouse hepatocytes	↑	AMPK ↑ → mTORC1 ↓ → autophagy ↑ → lipophagy ↑ → FAO ↑	[164]
PPARγ	Cannabinoid	HepG2	↑	eIF2α → TRIB3 → *Pparγ* gene ↑ → autophagic flux ↑ → apoptosis ↑ → HCC ↓ AMPK↑	[161]
	Pioglitazone	AML12, HFD-fed mice	↑	PPARγ activation ↑ → autophagy related genes *Atg7*, *Lc3*, & *Lal* ↑ → autophagy ↑ → lipolysis & FAO ↑ → hepatic steatosis ↓	[162]
	As_2_O_3_	Rat offspring liver	↑	PPARγ → ROS-mediated autophagy ↑ → As_2_O_3_ induced hepatotoxicity ↓	[163]
LXRα	GW3965 TO901317	Hepatocytes, HFD mouse liver, HepG2	↓	*let7a2* & *miR34a* genes ↑ → *Atg4B* & *Rab8B* genes ↓ → AP-lysosome fusion ↓ → lipophagy ↓ → FAO ↓ → hepatic steatosis ↑	[172]
HNF4α	-	HFD-fed mice	↑	HFD → miR214-3p gene ↑ → HNF4α-driven *Ulk1* mRNA ↓ → autophagy ↓	[192]
REV-ERBα	-	Zebrafish liver Mouse liver	↓	REV-ERBα → *Ulk1* gene ↓ Autophagy → degradation of circadian proteins BMAL1, CLOCK, REV-ERBα, and CRY1 ↑	[201,202]
RORα	-	*Rorα* LKO mice	↑	RORα → *Atp6v1g1* gene ↑ → lysosomal acidification ↑ → autophagy ↑	[205]
ERRα	-	Macrophage, HepG2, mouse liver	↑	AMPK/SIRT1 activation → *Errα* gene ↑ → *Atg5*, *Becn1*, *Atg16l1*, *Lc3b*, *& Ambra1* genes → AP ↑ ERRα → *Ulk1* gene ↑ → FUNDC1-LC3B-II interaction ↑ → mitophagy ↑	[72,211]
SHP	FGF19	Mouse liver, *Sh^−/−^* mice *Lsd1^−/−^* mice	↓	Feeding or FGF19 → FXR-SHP-LSD1 interaction ↑ → disrupting CREB-CRTC2 complex → autophagy related genes ↓ → autophagy ↓	[216]
		HSC	↓	*Shp* knockdown → autophagy related genes ↑ → autophagy ↑ → fibrotic-related genes ↑ → fibrosis ↑	[217]

E2, Estrogen; AMPK, AMP-activated protein kinase; mTOR, mammalian target of rapamycin; HK2, hexokinase 2; ULK1, unc-51-like kinase 1; FFA, free fatty acid; FAO, fatty acid oxidation; ROS, reactive oxygen species; CaMKK2, calcium/calmodulin dependent protein kinase kinase 2; FUNDC1, Fun14 domain containing 1; DAPK2, death associated protein kinase 2; CRTC2, CREB regulated transcription coactivator 2; Fgf21, fibroblast growth factor 21; MTF-1, metal regulatory transcription factor 1; FS DKO, Fxr/Shp double knockout; FS LDKO, liver-specific Fxr/Shp double knockout; OCA, obeticholic acid; UDCA, ursodeoxycholic acid; EtOH, ethanol; LPS, lipopolysaccharide; APAP, acetaminophen; HSC, hepatic stellate cell; eIF2α, eukaryotic translation initiation factor 2α; TRIB3, Tribbles pseudokinase 3; HCC, hepatocellular carcinoma; Lal, lipase A, lysosomal acid type; HFD, high-fat diet; ↑, increase; →, promote; ↓, decrease.

## Data Availability

Not applicable.
